# Association of internal smoking dose with blood DNA methylation in three racial/ethnic populations

**DOI:** 10.1186/s13148-018-0543-7

**Published:** 2018-08-23

**Authors:** Sungshim L. Park, Yesha M. Patel, Lenora W. M. Loo, Daniel J. Mullen, Ite A. Offringa, Alika Maunakea, Daniel O. Stram, Kimberly Siegmund, Sharon E. Murphy, Maarit Tiirikainen, Loïc Le Marchand

**Affiliations:** 1 Department of Preventive Medicine, Norris Comprehensive Cancer Center, Keck School of Medicine, University of Southern California, 1450 Biggy Street, NRT 1509G, Los Angeles, CA 90033 USA; 2 Epidemiology Program, University of Hawaii Cancer Center, 701 Ilalo Street, Honolulu, HI 96813 USA; 3 Department of Biochemistry and Molecular Biology, Keck School of Medicine, University of Southern California, Los Angeles, CA 90032 USA; 4 University of Hawaii John A. Burns School of Medicine, Honolulu, HI 96813 USA; 5 Masonic Cancer Center, University of Minnesota, Minneapolis, MN 55455 USA

**Keywords:** Smoking, Nicotine equivalents, DNA methylation, Japanese Americans, Native Hawaiians, Whites

## Abstract

**Background:**

Lung cancer is the leading cause of cancer-related death. While cigarette smoking is the primary cause of this malignancy, risk differs across racial/ethnic groups. For the same number of cigarettes smoked, Native Hawaiians compared to whites are at greater risk and Japanese Americans are at lower risk of developing lung cancer. DNA methylation of specific CpG sites (e.g., in *AHRR* and *F2RL3*) is the most common blood epigenetic modification associated with smoking status. However, the influence of internal smoking dose, measured by urinary nicotine equivalents (NE), on DNA methylation in current smokers has not been investigated, nor has a study evaluated whether for the same smoking dose, circulating leukocyte DNA methylation patterns differ by race.

**Methods:**

We conducted an epigenome-wide association study (EWAS) of NE in 612 smokers from three racial/ethnic groups: whites (*n* = 204), Native Hawaiians (*n* = 205), and Japanese Americans (*n* = 203). Genome-wide DNA methylation profiling of blood leukocyte DNA was measured using the Illumina 450K BeadChip array. Average β value, the ratio of signal from a methylated probe relative to the sum of the methylated and unmethylated probes at that CpG, was the dependent variables in linear regression models adjusting for age, sex, race (for pan-ethnic analysis), and estimated cell-type distribution.

**Results:**

We found that NE was significantly associated with six differentially methylated CpG sites (Bonferroni corrected *p* < 1.48 × 10−7): four in or near the FOXK2, PBX1, FNDC7, and FUBP3 genes and two in non-annotated genetic regions. Higher levels of NE were associated with increasing methylation beta-valuesin all six sites. For all six CpG sites, the association was only observed in Native Hawaiians, suggesting that the influence of smoking dose on DNA methylation patterns is heterogeneous across race/ethnicity (*p* interactions < 8.8 × 10−8). We found two additional CpG sites associated with NE in only Native Hawaiians.

**Conclusions:**

In conclusion, internal smoking dose was associated with increased DNA methylation in circulating leukocytes at specific sites in Native Hawaiian smokers but not in white or Japanese American smokers.

**Electronic supplementary material:**

The online version of this article (10.1186/s13148-018-0543-7) contains supplementary material, which is available to authorized users.

## Background

Globally, lung cancer is the second most common cancer, after prostate and breast cancer in men and women, respectively. It is also the most common cause of cancer-related death. Approximately 90% of lung cancer cases are ever smokers; however, only 11–24% of smokers will develop lung cancer in their lifetime [1]. Moreover, for the same quantity smoked, compared to whites, Native Hawaiians have been found to have a ~ 50% higher risk of lung cancer, whereas Japanese Americans have been shown to have a ~ 25% lower risk of disease [2, 3]. The lower risk of lung cancer in Japanese Americans can in part be explained by their slower nicotine metabolism (measured by CYP2A6 activity), which has been shown to influence smoking intensity (measured by biomarkers of internal smoking dose) resulting in a lower exposure to tobacco carcinogens [4, 5]. However, the higher risk of lung cancer in Native Hawaiians is inconsistent with their smoking intensity and nicotine metabolism rate, measured by CYP2A6 activity (the ratio of total trans-3′-hydroxycotinine over total cotinine), which is intermediate between that of whites and Japanese Americans. To date, this higher risk in Native Hawaiians is not explained by other known lung cancer risk factors.

DNA methylation of CpG sites is one of the most commonly studied epigenetic modification, and DNA methylation microarrays are the most common method to characterize the epigenome [6] in population studies. Multiple epigenome-wide association studies (EWAS) of smoking status have been conducted using DNA from blood leukocytes [7–19]. The most recent and largest study (*n* = 15,907) comparing current to never smokers found that 2623 CpG sites in 1405 genes were differentially methylated (Bonferroni corrected *p* = 1.48 × 10^−7^) [17]. The strongest association was with CpG sites located in the aryl-hydrocarbon receptor repressor (*AHRR*) gene, coagulation factor II (thrombin) receptor-like 3 (*F2RL3*) gene, G protein-coupled receptor (*GPR15*) gene, alkaline phosphatase genes, placental-like (*ALPPL2*) gene, and in genetic regions in 2q37.1 and 6p21.33 [18]. Hypomethylation of cg05575921 located in intron 3 of *AHRR* is one of the most frequently replicated findings and may serve as a marker for current smoking status, cumulative amount smoked (smoking pack-years), smoking dose (cigarettes per day [CPD]), and time since quitting [7–16]. However, the literature investigating the influence of self-reported smoking dose (assessed by CPD) or internal dose (assessed by cotinine measurement) is limited to four and three studies, respectively. To our knowledge, no EWAS of nicotine equivalents (NE) has been conducted. NE is a more comprehensive measure of internal smoking dose than other smoking metabolites, such as cotinine, as it is the sum of the major metabolites of nicotine: total cotinine (nmol/mL), total nicotine (nmol/mL), and total trans-3′-hydroxycotinine (3-HC, in nmol/mL), which includes their glucuronides, accounting for ~ 80% of nicotine uptake [20]. Thus, unlike cotinine, NE accounts for the variations in nicotine metabolism across multiethnic populations [5, 21]. Moreover, no study has evaluated whether peripheral blood DNA methylation patterns differ by race/ethnicity for the same NE. Characterization of these differences might be enlightening since racial/ethnic groups have been found to have variations in nicotine uptake per cigarette [5]. The differential impact of smoking dose on the epigenome may in part contribute to the ethnic variations in smoking-related lung cancer risk.

We conducted the first EWAS of NE in three populations with different risks for lung cancer to identify potential mechanisms for the differences in smoking-related disease risks. We hypothesized that an increase in smoking dose will be associated with differential methylation of epigenetic regions in blood leukocyte DNA and that for the same dose, the associations may vary across race/ethnicity. We also evaluated potential biological pathways based on the genes involved in our top associations.

## Results

Table 1 presents the characteristics of the 612 Japanese Americans, Native Hawaiians, and white participants enrolled in this study. Native Hawaiians were slightly younger than Japanese Americans and whites (mean age = 57 years versus 62 years, respectively). The distribution of males and females was very similar as an equal number of men and women were targeted for recruitment. Native Hawaiians were heavier (body mass index [BMI] = 29.3 kg/m^2^) followed by whites (26.6 kg/m^2^) and Japanese Americans (25.5 kg/m^2^). Whites reported smoking the most CPD and had the highest NE (CPD = 26.3 and NE = 55.2 nmol/ml), followed by Native Hawaiians (CPD = 21 and NE = 50.3 nmol/ml) and Japanese Americans (CPD = 19 and NE = 35.0 nmol/ml). Lifetime smoking quantity was lowest in Native Hawaiians (42.4 pack-years), which is expected as Native Hawaiians on average were younger at time of urine collection, followed by Japanese Americans (44.7 pack-years) and whites (56.8 pack-years). We found that the cell type by race/ethnicity differed for CD8, T cells, B cells, natural killer cells, and monocytes (*p* values < 0.005) (Table 1). Also, heterogeneity in the relationship between NE and cell types by race/ethnicity was also detected. Specifically, NE was inversely associated with natural killer cells in Native Hawaiians and Japanese Americans, inversely associated with monocytes in whites, and positively associated with B cells in Native Hawaiians (Additional file 1: Table S1). Among 35 never smokers (11 Japanese Americans, 12 Native Hawaiians, and 12 whites) who were included in only the EWAS of smoking status, the distribution of males and females were ~ 50% in each racial/ethnic group. The Japanese American and Native Hawaiians were older (69 and 67 years of age, respectively) than their current smoking counterparts, whereas the white never smokers were similar in age as their current smoking counterpart (63 years).

**Table 1:** Demographic characteristics of study population current smokers at time of biospecimen collection stratified by race/ethnicity

	Japanese Americans	Native Hawaiians	Whites
*N*	203	205	204
Age (years), mean (SD)	61 (6.95)	57 (12.82)	62 (6.85)
Gender, *N* (%)			
Males	101 (49.7)	101 (49.3)	101 (49.5)
Females	102 (50.3)	104 (50.7)	103 (50.5)
BMI (kg/m^2^), mean (SD)	25.53 (4.71)	29.29 (7.05)	26.61 (5.78)
Pack-years, mean (SD)	44.68 (17.95)	42.44 (24.15)	56.82 (24.53)
CPD, mean (SD)	19.93 (7.45)	21.15 (9.5)	26.32 (11.38)
CYP2A6 activity, mean (SD)	0.85 (1.47)	1.08 (1.14)	1.43 (1.73)
NE (nmol/ml)	34.98 (27.78)	50.26 (41.49)	55.20 (48.86)
Cell types, mean (SD)			
CD8 cells**	0.03 (0.04)	0.05 (0.04)	0.02 (0.03)
CD4T cells	0.16 (0.07)	0.16 (0.06)	0.16 (0.08)
Natural killer cells*	0.06 (0.04)	0.06 (0.04)	0.04 (0.03)
B cells**	0.08 (0.04)	0.08 (0.03)	0.06 (0.03)
Monocytes	0.05 (0.04)	0.05 (0.03)	0.06 (0.04)
Granulocytes*	0.67 (0.12)	0.66 (0.11)	0.69 (0.12)

*P value for trend < 0.005, indicating significant ethnic differences

**P value for trend < 0.0005, indicating significant ethnic differences

Additional file 1: Table S2 presents a list of probes (*n* = 55) that have been found in at least five EWAS of blood leukocytes to be differentially methylated by smoking status (current versus never). This table also presents the parameter estimates and *p* values from the association tests for these probes with smoking status, NE, and CPD within our study population. Among the ten most frequently replicated (> 10 studies) probes (marked with an asterisk (*)), all ten were also found to be associated with another measure of smoking quantity pack-years, CPD, or cotinine (from at least one EWAS or candidate-probe analyses). In our EWAS of smoking status (*n* = 612 current smokers versus 35 never smokers), after adjustments for age, sex, race/ethnicity, and cell type, 24 of the previously 55 replicated probes were associated with smoking status at a Bonferroni significance level (*p* = 1.48 × 10^−7^). Among our three most significant hits, cg0591221 and cg21566642 in 2q37.1 (*p* = 1.95 × 10^−67^ and 4.4 × 10^−67^) and cg05575921 in *AHRR* (*p* = 7.99 × 10^−67^), current smoking status was associated with decreasing methylation beta values. For all 24 replicated probes, we found that the associations had greater statistical significance in whites than the other two racial/ethnic groups, despite a similar sample size across the three ethnic populations (e.g., for *AHRR*, cg05575921 *p* = 4.3 × 10^−31^ for whites, *p* = 2.9 × 10^−20^ for Japanese Americans, and *p* = 7.1 × 10^− 19^ for Native Hawaiians).

**Fig. 1:**
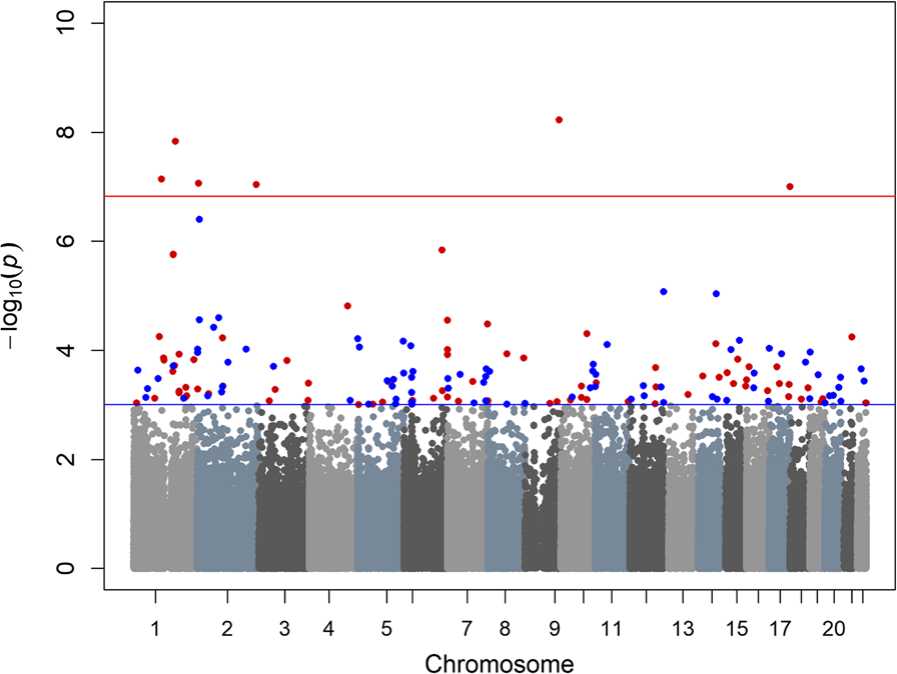
Manhattan plot of the *p* values for the association for nicotine equivalents with DNA methylation. *Red line: Bonferroni corrected *p* value *p* = 1.48 × 10^−7^, blue line: *p* = 1 × 10^−3^. **Red dots indicate that the parameter estimate is in the positive  direction, and blue dots indicate parameter estimates that are in the negative  direction

**Fig. 2:**
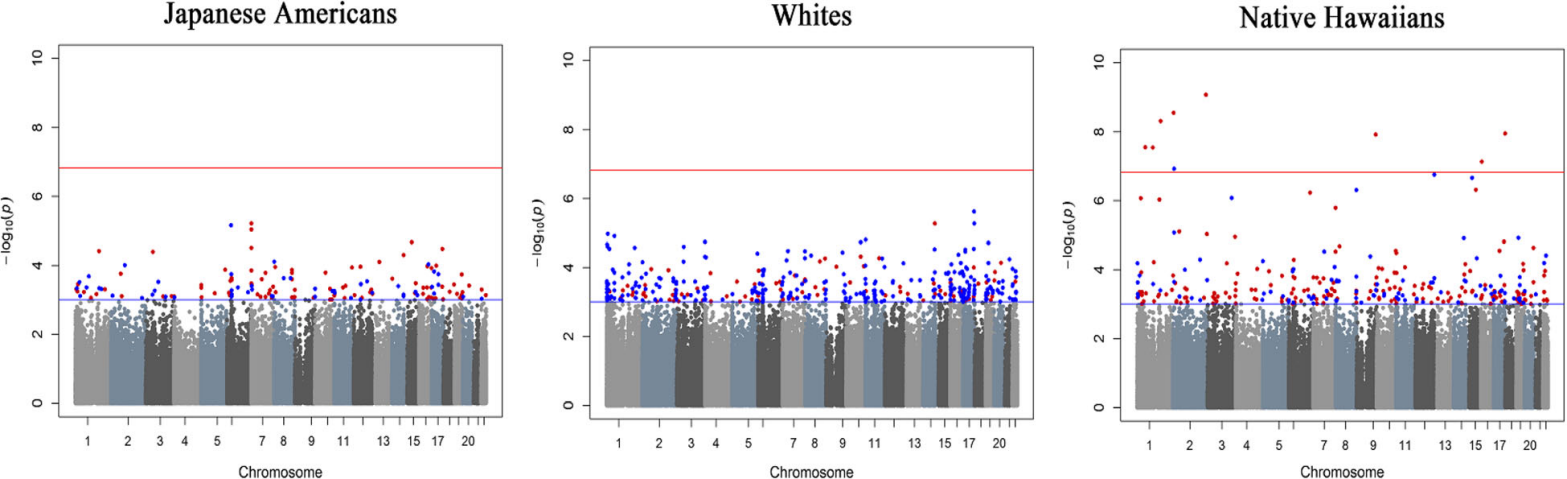
Manhattan plots of the *p* values for the association for nicotine equivalents with DNA methylation, stratified by race/ethnicity. *Red line: Bonferroni corrected *p* value *p* = 1.48 × 10^−7^, blue line: *p* = 1 × 10^−3^. **Red dots indicate that the parameter estimate is in the positive direction and blue dots indicate parameter estimates that are in the negative  direction

**Fig. 3:**
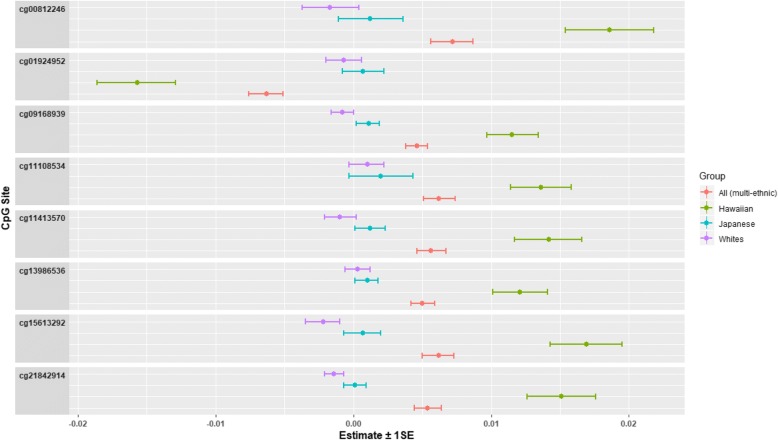
Forest plots of the associations for nicotine equivalents with DNA methylation, stratified by race/ethnicity

In our pan-ethnic EWAS of smoking dose in smokers, after adjusting for age, sex, race/ethnicity, and cell type, we found that NE was associated with six differentially methylated probes (Bonferroni corrected *p* < 1.48 × 10^−7^) (Fig. 1). For all six probes, higher levels of NE were associated with increasing methylated beta-values (Table 2). These six CpG probes were mapped in or near the *FOXK2*, *PBX1*, *FNDC7*, and *FUBP3* genes and two in non-annotated regions. Associations with these six probes also showed statistically significant interactions with race, where the associations were only statistically significant in Native Hawaiians (*p* < 1.1 × 10^−8^). These differences indicate that Native Hawaiians may have greater differential DNA methylation patterns in relation to smoking dose, compared to whites and Japanese Americans (*p* interaction < 8.8 × 10^−8^) (Figs. 2 and 3). In ethnic-specific EWAS (Additional file 1: Table S4), higher NE was associated with increasing methylation beta-values of two additional probes, cg00812246 in *BSND* and cg01924952 in 2p25.1 (*p* < 1.2 × 10^−7^), in only Native Hawaiians (Table 2). When investigating the association between CPD and DNA methylation, no significant pan-ethnic or ethnic-specific associations were detected (*p* < 1.48 × 10^−7^). In our data, NE and CPD were found to have a modest, but statistically significant correlation (Pearson’s *r* = 0.12; *p* < 0.001).

**Table 2:** Association between NE and DNA methylation (*p* value < 1.48 × 10^−7^ from pan-ethnic and/or ethnic-specific analyses)*

Probe	Chr	Cytoband	Mapinfo	CGI region	Ref_Gene	Overall		Whites		Japanese Americans		Native Hawaiians	
						Estimate	*P* value	Estimate	*P* value	Estimate	*P* value	Estimate	*P* value
cg11413570	1	1p13.3	109260678	S_Shore	FNDC7	0.0056	7.15E−08	− 0.0010	0.3870	0.0012	0.2774	0.0142	2.84E−08
cg00812246	1	1p32.3	55464868		BSND	0.0071	5.35E−06	− 0.0017	0.4165	0.0012	0.6007	0.0186	2.78E−08
cg09168939	1	1q23.3	164652019		PBX1	0.0046	1.46E−08	− 0.0008	0.3237	0.0011	0.2119	0.0115	4.82E−09
cg01924952	2	2p25.1	8627933		–	− 0.0063	3.96E−07	− 0.0006	0.6050	0.0007	0.6307	− 0.0157	1.19E−07
cg11108534	2	2p25.2	5584457		–	0.0062	8.65E−08	0.0010	0.4293	0.0020	0.3853	0.0136	2.79E−09
cg15613292	2	2q37.1	232481541		–	0.0062	9.04E−08	− 0.0022	0.0736	0.0007	0.6079	0.0169	8.27E−10
cg13986536	9	9q34.2	133456512	S_Shore	FUBP3	0.0050	5.84E−09	0.0003	0.7671	0.0010	0.2783	0.0121	1.19E−08
cg21842914	17	17q25.3	80545869	S_Shore	FOXK2	0.0054	9.86E−08	− 0.0014	0.0514	0.0001	0.9237	0.0151	1.10E−08

*In order by chromosome

We found that none of the 55 well-replicated probes (≥ 5 studies) linked with smoking status were associated with NE at a Bonferroni significance level. However, eight of the 55 were modestly associated with NE (*p* < 0.05) (Additional file 1: Tables S2 and S3). Six of these eight were among the 10 most frequently replicated probes (> 10 studies) with current smoker status; the other two probes, cg25949550 in contactin-associated protein-like 2 (*CNTNAP2*) and cg02657160 in coproporphyrinogen oxidase (*CPOX*), were previously found to be associated with smoking status and with cotinine in ever smokers [22]. The most statistically significant association being cg05575921 in *AHRR* (regression coefficient = − 0.0154; *p* = 6 × 10^−5^). Among current smokers, no significant associations between CPD and DNA methylation were detected in these 55 probes (*p* > 1.48 × 10^−7^). We found three of the 55 well-replicated probes were modestly associated with CPD (*p* < 0.05): cg13193840 in 2q37.1, cg19859270 in GPR15, and cg24090911 in *AHRR* (*p* = 0.018, 0.035, and 0.047, respectively).

A gene list based on differentially methylated probes associated with NE (*p* < 10^−3^) (Additional file 1: Tables S3 and S4) was used for pathway analysis. Based on Ingenuity Pathway Analysis (IPA) of the genes identified to have overall pan-ethnic or ethnic-specific association with NE, the vast majority of genes were identified as playing a role in cancer (86% of 117 genes for the pan-ethnic; 90% of the 278 genes for whites; 95% of the 108 genes for Japanese Americans; and 92% of the 206 genes for Native Hawaiians). Interestingly, we found that there was no overlap of genes or probes across all three ethnicities. Only four genes overlapped in whites and Japanese Americans, two overlapped in Japanese Americans and Native Hawaiians, and 10 in whites and Native Hawaiians (Additional file 2: Figure S1).

The IPA analysis from the ethnic-specific gene lists found that the top three canonical pathways in whites were reelin signaling in neurons (*p* = 0.004), thrombin signaling (*p* = 0.01), and tight junction signaling (*p* = 0.01) (Additional file 1: Table S5). In Native Hawaiians, the canonical pathways were dermatan sulfate degradation (Metazoa) (*p* = 0.02), netrin signaling (*p* = 0.02), and γ-glutamyl cycle (*p* = 0.02)). In Japanese Americans, the canonical pathways were ephrin B signaling (*p* = 2.2 × 10^−5^), IL-1 signaling (*p* = 7.1 × 10^−5^), and relaxin signaling (*p* = 0.001).

**Fig. 4:**
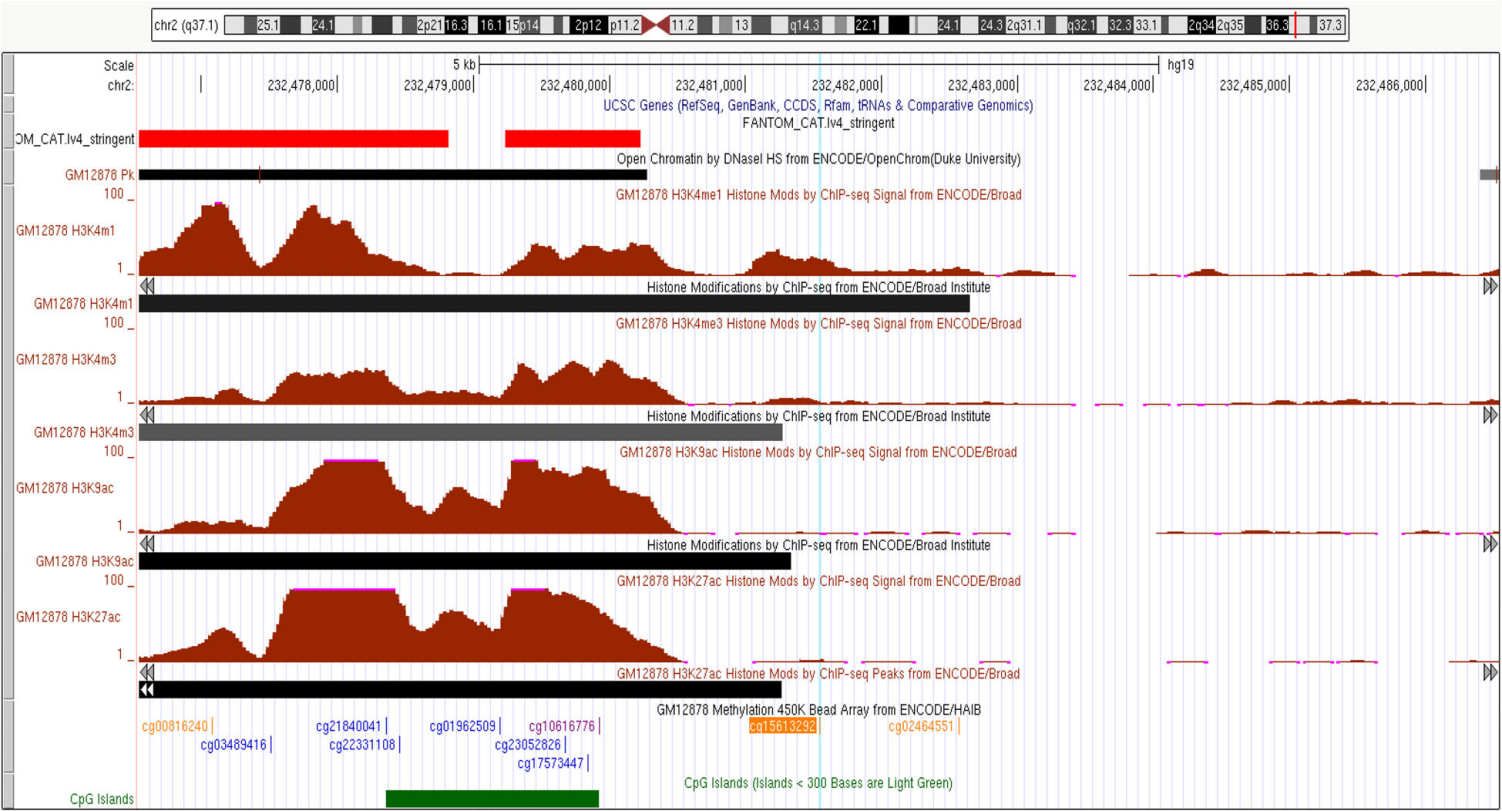
Regulatory features of cg15613292 in 2q37.1

**Fig. 5:**
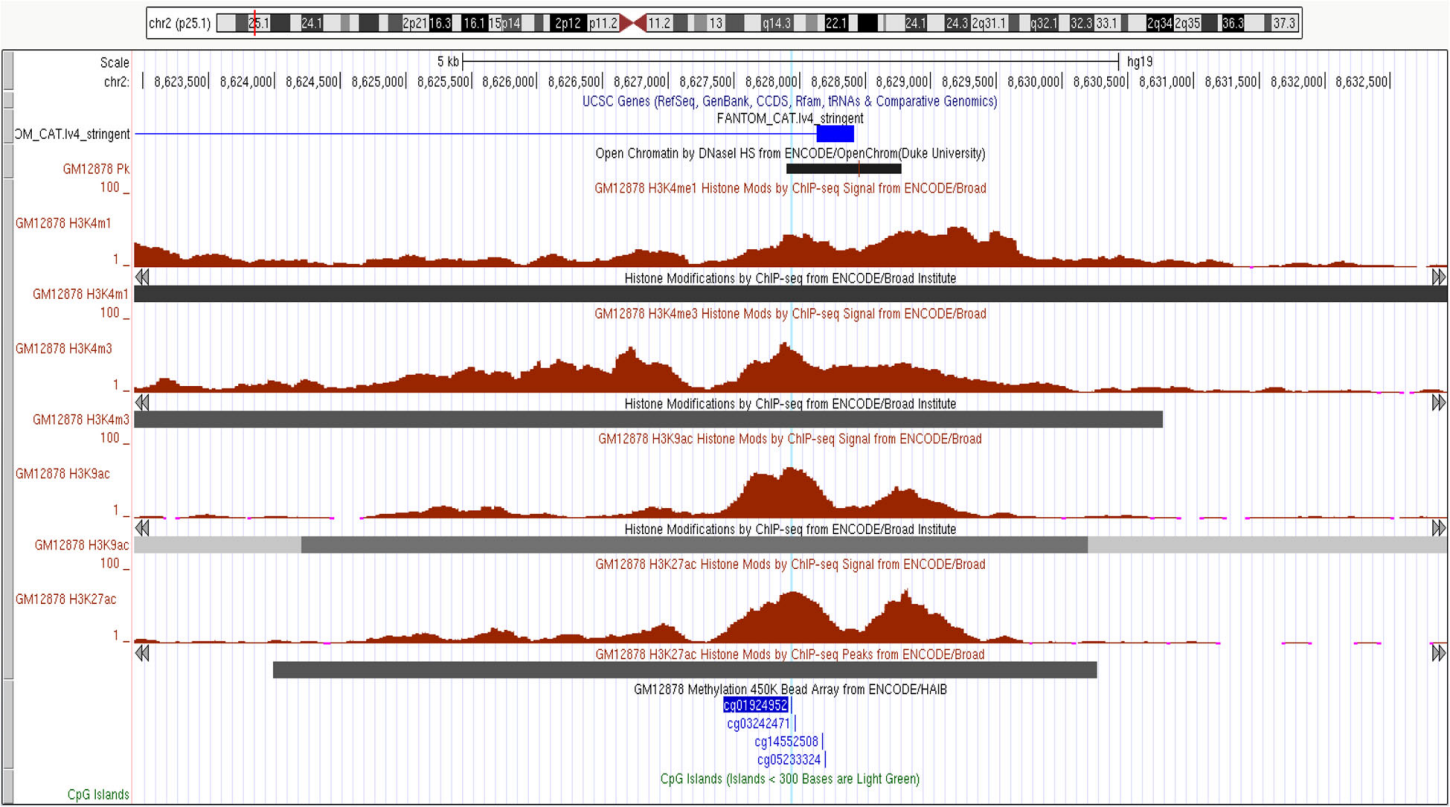
Regulatory features of cg01924952 in 2p25.1

To gain insight into the possible functional role of the top eight significantly differentially methylated probes associated with NE from either our pan-ethnic or ethnic-specific analysis (*p* < 1.48 × 10^−7^), we utilized ENCODE publicly available epigenomic data [23] (Figs. 4 and 5 and Additional file 2: Figures S2–S7). None of the eight top probes are located within CpG islands. Three probes (cg15613292, cg21842914, and cg13986536) are located within a CpG shore, just downstream (< 2 Kb) of a CpG island (Fig. 4 and Additional file 2: Figures S2–S3). Three of the six top probes (cg15613292, cg01924952, and cg13986536) are in putative transcriptional regulatory domains in the GM12878 cell line (B-lymphocytes) (Figs. 4 and 5 and Additional file 2: Figure S2). Cg21842914 is in a putative transcriptional regulatory domain in CD20+ cells (B cells) and CD14+ cells (monocytes) (Additional file 2: Figure S3). Cg15613292 (2q37.1) is located in an intergenic region within 1 kb of a large enhancer and the closest gene (C2orf57) is located > 22 Kb away (Fig. 4). Cg13986536 (9q34.11) is located in intron 1 of the *FUBP3* gene near a region carrying DNAse hypersensitivity marks as well as histone promoter and enhancer marks (Additional file 2: Figure S2), possibly functioning to regulate expression of the *FUBP3* gene, or an adjacent uncharacterized long noncoding RNA (LOC100272217). Cg01924952 (2p25.1) is located in a putative enhancer in a large intergenic region, with the closest gene (> 150 Kb) encoding for a long non-coding RNA, *LINC00299* (Fig. 5).

## Discussion

This is the first study to evaluate the association of a NE with DNA methylation in circulating leukocytes across multiple racial/ethnic populations with different smoking-related lung cancer risk. Overall, we found similar methylation patterns associated with smoking status as in other studies. Higher NE levels were statistically significantly associated with an increase in DNA methylation *β* values (Bonferroni correction *p* < 1.48 × 10^−7^) in Native Hawaiians, but not in whites or Japanese Americans. Our findings suggest that Native Hawaiians have greater differential DNA methylation patterns, compared to whites and Japanese Americans, in relation to internal smoking dose, as measured by NE.

DNA methylation in relation to smoking status has been one of the most frequently studied epigenetic modifiers. There are > 2000 probes that have been found associated with measures of smoking (e.g., status, pack-years, time since quitting, dose) [18]. Among the four studies that have conducted EWAS of smoking dose [8, 10, 13, 22], all quantified dose by self-reported CPD and three of the four also conducted an EWAS of cotinine levels [10, 13, 22]. In addition, all four studies included both never and ever smokers in the discovery stage. In contrast, our study used NE, which is a better marker of smoking dose than CPD and cotinine, as it accounts for the variations of dose per cigarette from differences in smoking behavior (e.g., depth of inhalation) and/or nicotine metabolism present across multiethnic populations [5, 20]. Our study of NE was restricted to current smokers, as opposed to including both never and ever smokers. While the inclusion of never smokers improves the power to detect an association, such results also capture the associations with smoking status and not just with smoking dose.

In our EWAS of NE among current smokers, the top eight probes associated with NE were not among the probes most frequently associated with smoking status. We found that in seven of our top eight probes, higher NE was associated with an increase in methylation levels. These results are interesting, as previous epigenetic studies of smoking found that many markers associated with smoking status are found to be hypomethylated in current smokers compared to never or former smokers. However, importantly, smoking dose on the epigenome in only current smokers has not been systematically investigated. There has been only one other EWAS of smoking dose (self-reported CPD and cotinine) in current smokers [22]. Here, the investigators found that when including both never and ever smokers (*n* = 1000), 40 probes had a dose-response relationship with cotinine at a Bonferroni significance level (*p* = 1.13 × 10^−7^) [22]. All 40 probes were previously associated with smoking status [22]. When restricting the analysis to only current smokers (*n* = 176), only five of the 40 probes remained associated (*p* for non-linearity < 0.05) and one probe cg22132788 at *MYO1G* had a dose-response relationship in a different direction. The loss of statistical significance may be the result of the reduction in sample size or suggest that methylated probes associated with smoking status may not be same as those affected by smoking dose in current smokers. Similarly, in our EWAS of NE in current smokers, we found that none of the 55 most frequently replicated smoking status probes were associated with NE at *p* < 10^−7^ and only eight of these were modestly associated with NE (*p* < 0.05).

Moreover, in our multiethnic study of 612 current smokers (~ 200 samples per race/ethnicity), the epigenome of Native Hawaiians appeared to be differentially sensitive to the effects of internal smoking dose (Fig. 3), with eight probes mapping in or near six genes: *FNDC7*, *BSND*, *PBX1*, *FUBP3*, *FOXK2*, and three in intergenic regions. These eight probes have not been previously identified as differentially methylated probes in relation to smoking dose (including never smokers). The majority of the previous EWAS of smoking dose were conducted in populations of European descent, whereas a small minority were conducted in populations of non-European descent (such as African or South Asian descent) [10, 11, 15, 17, 24], and no study included Native Hawaiians. No prior EWAS of smoking dose were conducted in populations of non-European descent. In the two multiethnic EWAS of smoking status, one study detected an interaction by race (South Asian and whites) with the DNA methylation beta values for cg05575921 (in *AHRR*) (*p* = 2.0 × 10^−3^) among 36 current smokers, where the median methylation beta values were higher in South Asian smokers than white smokers, even after adjusting for CPD [24]. The other study found that differential methylation of cg05575921 by smoking status was more significant in whites than in African Americans (*p* values = 1.45 × 10^−62^ vs. 4.61 × 10^−16^) [17]. However, the authors stated that this difference was likely a result of the smaller sample size in African Americans and the *p* value for interaction was not shown [17]. In our current study, the sample size for each of the three ethnicities was similar (*n* ~ 200). Variations of cell type distribution across race/ethnicity may also have contributed the differential impact of NE on methylation across populations. A study of 20 smokers and 14 nonsmokers found that smoking may differentially affect the epigenome by cell type lineage [25]. For instance, for cg05575921 in *AHRR*, the reduction in methylation levels in smokers (compared to non-smokers) was greatest in granulocytes and monocytes, followed by B cells and not observed in T cells [25].

Differential methylation of probes in *AHRR* is one of the most commonly replicated regions across EWAS of smoking-related phenotypes. Cg05575921 has served as marker of smoking status [26] and second-hand smoking [27] and has been associated with smoking quantity (pack-years) in ever smokers and quit time in past smokers [14, 28]. Differential methylation of cg05575921 has been found associated with lung cancer, where cases had lower DNA methylation beta values than controls, even after adjusting for age, sex, smoking status, and pack-years [29, 30]. AHRR serves as a negative feedback regulator of aryl hydrocarbon receptor (*AHR*), which plays a critical role in the metabolic activation of polycyclic aromatic hydrocarbons (PAHs), found in cigarette smoke. AHHR competes with AHR for binding with the aryl nuclear receptor translocator (*ARNT*). We recently showed that cg05575921 lies right adjacent to a tobacco smoke-inducible enhancer [31]. Hypomethylation of cg05575921, as well as the transcriptional activation of *AHRR*, was replicated in vitro by treating A549 cells with cigarette smoke concentrate [31]. Thus, at least in some cases, changes in DNA methylation appear to be the result of changes in the activation of regulatory elements. The gain or loss of factors interacting with the DNA in response to environmental cues might alter the accessibility of CpGs to the maintenance DNA methyltransferase.

We found that three of the probes (cg13986536 in *FUBP3*, cg15613292 in 2q37.1, and cg21842914 in *FOXK2*) that were differentially methylated with smoking dose were located in CpG shores, just downstream (< 2 Kb) of CpG islands. All three are located in putative transcriptional regulatory domains based on their proximity to DNase hypersensitivity cluster and histone modification tracks in leukocytes. *FUBP3* is a member of a family of single-strand DNA binding proteins that bind to the far-upstream element (FUSE) of the *MYC* gene regulating its expression [32]. In a GWAS to study the stages of smoking progression, an intronic SNP (rs2304808) in *FUBP3* was associated with greater smoking tolerance (defined as “years from the age of daily smoking to the age when the heaviest smoking started”), independent of cigarette quantity [33]. The evaluation of methylation quantitative trait loci between this variant and cg13986536 is warranted. Cg15613292 is located in an intergenic region in 2q37.1, with the closest gene, *C2orf57* (also known as *TEX44*), more than 22 Kb away. However, two putative long non-coding RNA genes in the region (< 10 Kb away) were recently reported in the FANTOM5 project database [34]. Interestingly, the *p* interaction by race/ethnicity was most significant for cg15613292 (*p* < 10^−11^) in Native Hawaiians; a log unit increase in NE was associated with a statistically significant increase in methylation beta-values. In contrast, in whites, the same log unit increase in NE was associated with a suggestive decrease in methylation beta-values. In addition, this probe is 4466 bp away from cg00816240, which was previously found to be inversely associated with current smoking status in an EWAS comprised of mostly European descent individuals [17]. Cg00816240 is also located in an exon of one of the putative long non-coding RNA identified in the FANTOM5 project.

Based on data from ENCODE, the differentially methylated probe, cg21842914 located in intron 8 of the *FOXK2* gene, is not located in a transcriptional regulatory region in GM12878 cells (B-lymphocytes) but is in a regulatory region for CD20+ and CD14+ cells based on histone modification levels. This genetic region has been found to be differentially methylated by smoking status in two other EWAS studies [17, 35]. In an EWAS of 31 current and 39 never Korean smokers to identify differential methylated regions (as opposed to only probes), the investigators found that probes within 80,545,020-80,545,869 bp of *FOXK2* were differentially methylated by current smoking status (cg21842914 is located at 80,545,869) [35]. Also, EWAS of current smoking status in primarily whites and some African Americans (*n* = 15,907) found that four probes in *FOXK2* (cg20412356, cg20173014, cg02094337, and cg16375265) were modestly associated with current smoking status (*p* < 0.0015) [17]. The most significant of the four probes, cg02094337 (*p* = 2.3 × 10^−6^), is 3751 bp away from cg21842914. We found that for cg21842914, a log unit increase in NE was associated with increasing methylated beta-values in Native Hawaiians and a modest decrease in methylated beta-values in whites. *FOXK2* is part of the family of forkhead transcription factors that control cell cycle and represses transcription of the cyclin-dependent kinase inhibitor gene p21 [36]. Thus, at least four of the top eight CpGs lie in or near regulatory regions that might be affected by environmental exposures such as smoking.

Based on the IPA analysis, the most significant of the implicated canonical pathways is ephrin B signaling, which has been shown to play an important role in lung cancer [37]. The ephrin type-B receptor 4 (EPHB4) tyrosine kinase has been shown to be overexpressed in lung tumors [38], and mutations in *EPHB4* identified in lung tumors can induce cellular proliferation and migration [38]. Ephrin type-B receptor 3 (EphB3) expression has been found to be higher in non-small cell lung cancer as well as associated with metastasis [39].

This study had some limitations worth noting. The generalizability of our findings is limited as we assessed methylation changes in leukocytes and not in the target tissue [40]. Also, we adjusted for predicted cell type measures with an algorithm that may not be valid for all racial/ethnic groups. In addition, we carried out IPA analysis using the genes nearest to the affected CpGs; however, it may not always be the nearest target gene whose expression is affected, in particular, if the CpG lies in or near the enhancer [31]. Lastly, a slightly more comprehensive measure of internal smoking dose, total nicotine equivalents (the sum of NE and nicotine N-oxide), was not measured in this study. However, on average, nicotine N-oxide only accounts for < 5% of the total nicotine equivalents. Strengths of this study include the multiethnic composition and relatively large sample size of current smokers, the use of a biomarker of internal smoking dose, and the ability to adjust for a range of potential confounders.

## Conclusions

In conclusion, we identified eight differentially methylated probes in relation to internal smoking dose. These associations were primarily found in Native Hawaiians, suggesting that smoking may impact the epigenome differentially by race/ethnicity. Further investigation on how these methylation sites may influence transcriptional regulation and modify function within a biologic context is warranted. Our findings suggest that smoking dose may differentially impact the epigenome by ethnicity, which may provide insights into the mechanisms that contribute to the observed differences in lung cancer risk across these three populations.

## Methods

### Study population

The primary analysis was conducted in a study of only current smokers in Hawaii. The study has been described in detail previously [4, 41]. In brief, 612 smokers were randomly identified from either the Hawaii component of the Multiethnic Cohort study (MEC) (87%) or control groups of population-based case-control studies conducted in Hawaii (13%). The MEC is a prospective study of > 215,000 men and women of five racial/ethnic groups: African Americans, Japanese American, Native Hawaiian, Latinos, and whites, recruited from the state of Hawaii and from Southern California, primarily Los Angeles County, between 1993 and 1996 [42]. For the present study, eligible participants were identified among those who reported smoking at least 10 CPD on their respective study questionnaire, have had no previous history of cancer, and whose parents are both of Japanese or both of Caucasian ancestry or for whom one or both parents have any amount of Native Hawaiian ancestry. To confirm previously published associations between smoking status and DNA methylation, 35 randomly selected never smokers from the MEC (*n* = 12 Native Hawaiians, 11 Japanese Americans, and 12 whites) were included in the EWAS of smoking status. Never smoking status was identified by self-report at time of biospecimen collection.

### Data collection

For this study, all interviews were conducted in the participants’ home. Information was collected on lifetime tobacco and alcohol use and lung cancer-related occupational exposures. Participants were also instructed on how to keep a food record and a diary of all medications and dietary supplements for the 3 days preceding a 12-h overnight urine collection and a blood draw. Urine collection began between 5 and 9 pm for a period of 12 h which included all urine passed through the night and the first morning urine. The urine was kept on ice until processing, which occurred within 4 h of the last sample. Aliquots were subsequently stored in a − 80 °C freezer until analysis.

### Laboratory analysis and quality control

Nicotine equivalents (NE) comprised of total cotinine (nmol/mL), total nicotine (nmol/mL), and total trans-3′-hydroxycotinine (3-HC, in nmol/mL) which includes their glucuronides. The methodology to measure these biomarkers was previously reported [4]. In brief, total urinary nicotine, cotinine, and 3-HC were measured by gas chromatography/mass spectrometry. For total nicotine (free + nicotine N-glucuronide) and total cotinine (free + cotinine N-glucuronide) concentration, the samples were treated with base to cleave the glucuronide conjugates, and the nicotine and cotinine were quantified by gas chromatography/mass spectrometry analysis [43]. For total 3-HC (3-HC + its glucuronide), the sample was first treated with h-glucuronidase and then analyzed 3-HC by gas chromatography/mass spectrometry, as described previously [44]. The sum of these metabolites accounts for over 75% of nicotine and its metabolites [45] and has been used to quantify the majority of nicotine uptake.

### DNA methylation

DNA was extracted from buffy coat using the QIAamp DNA Blood Mini kit (Qiagen) and quantified using the Quant-iT Pico Green dsDNA Assay Kit (Invitrogen). DNA was normalized to 50 ng/ul and plated without regard to ethnicity. Five hundred nanograms of the extracted DNA was treated with bisulfite using EZ-96 DNA Methylation kit (Zymo Research, Orange, CA, USA), and 100–200 ng of bisulfite-converted DNA was hybridized onto the Infinium Human Methylation450 BeadChip (Illumina, San Diego, CA).

This array covers 485,512 loci, representing 96% of known CpG islands and 99% of RefSeq genes, with an average of 17 CpG sites per gene distributed across the upstream region of the transcription start sites (TSS) 1500, TSS200, 5′UTR, first exon, gene body, and 3′UTR.

After hybridization, washing, and single nucleotide extension steps, the BeadChips were scanned on the Illumina iScan and the intensities of the images recorded in .IDAT files. The probe intensities were processed using the minfi package in Bioconductor [46, 47], with fluorescence intensities normalized utilizing various internal controls that are present on the Methylation450 BeadChip. Within-sample normalization consisted of background correction, dye-bias correction to normalize any imbalance due to intensities measured in two color channels, and type I and II probe intensity normalization to correct for differences in range of signal intensity due to two probe types [48, 49]. The DNA methylation score for each CpG site is represented by a *β* value between 0 (fully unmethylated) to 1 (fully methylated) calculated from the fluorescence intensity ratio between methylated and methylated + unmethylated probes for each tested CpG. *β* values can easily be interpreted as the proportion of methylated DNA at a given locus.

Each chip included a randomly selected mix of racial/ethnic groups, sex, and smoking levels to minimize the impact of any batch effects. To avoid gender- and genotype-associated biases [50], non-autosomal CpG loci and loci positioned at a single nucleotide polymorphism (SNP) (dbSNP build 137) were excluded. This resulted in 337,542 probes being retained for the analysis.

### Cell-type calculations

Cell type proportions for CD4 T cells, CD8 T cells, B cells, natural killer cells, monocytes, and granulocytes were calculated using an online calculator [51, 52]. Adjusting for cell types was justified based on the known inflammation and immune response changes due to smoking [53].

### Statistical methods

Pearson partial correlation coefficients, adjusting for age, sex, and race/ethnicity, were assessed between CPD and NE. Median and interquartile ranges were used to describe the cell type data distribution. ANOVA was used to evaluate cell type distribution differences across the three populations. NE was not normally distributed and thus log transformed. To evaluate the association of log NE with DNA methylation *β* values, linear regression models were used. We adjusted for age, sex, race/ethnicity (in pan-ethnic analyses, i.e., all races combined), and estimated cell-types distribution. Other variables such as BMI were considered. Findings did not change with the inclusion of BMI and thus were not presented. All tests for heterogeneity of effects by race/ethnicity were conducted by including an interaction term between log-NE and race/ethnicity. For the pan-ethnic analyses, we did not use random effects model as our study sample was a sample of convenience and not a random cluster sampling from an ideal super population. Thus, the variance from such a random effects model would not properly define the degree of heterogeneity in the super population. Moreover, our conclusions as to whether heterogeneity in the effects of methylation exists across these three populations would be the same whether we used a fixed or random effects model. An EWAS of smoking status (*n* = 35 never vs 612 current) was conducted to confirm previously identified DNA methylation findings. Also, after analyses were completed, the significant probes were evaluated for poor mapping quality [54]; those considered to be poor were excluded.

### Bioinformatics analysis

Gene lists were generated based on proximity to the significant differentially methylated probes (*p* < 10^−3^). The Ingenuity Pathway Analysis software (IPA; Ingenuity® Systems, www.ingenuity.com) was used to analyze our gene lists to identify an enrichment for genes functioning in specific signaling pathways and biological mechanisms associated with smoking dose.

We also used the Encyclopedia of DNA Elements (ENCODE) Consortium data [23, 55] to determine if the significant probes were located in putative regulatory regions (e.g., DNase hypersensitivity and histone modification) in multiple cell types. We examined the regulatory features in human embryonic stem cells, an immortalized B-lymphocyte cell line (GM12878 cells within a 10 kb window of the eight CpGs), focusing on CpG islands, DNase hypersensitivity clusters and four histone modifications: histone 3 lysine 4 monomethylation (H3K4me1, a mark for poised or active enhancers), histone 3 lysine 4 trimethylation (H3K4me3, a promoter mark), histone 3 lysine 9 acetylation (H3K9ac, a mark found near transcription start sites), and histone 3 lysine 27 acetylation (H3K27ac, an active enhancer mark found at proximal and distal regions of transcription start sites). We also considered the regulatory features in CD20 and CD14 cell lines. The probes were considered as potential regulatory probes if they were in regions containing DNase hypersensitivity and/or histone modifications (H3K4me1, H3K4me3, H3K9ac, HeK27ac).

## Additional files

Additional file 1: **Table S1.** Results of association of NE with cell type among current smokers by ethnic group. Table S2 The association results for smoking status, NE and CPD with DNA methylation levels for 55 most frequently replicated probes previously found associated with smoking status. Table S3 Association between DNA methylation and NE (*p* < 1 × 10^−3^). Table S4 Significant association between NE and DNA methylation (*p* value < 1 × 10^−3^ for any ethnic group). Table S5 The top canonical pathways and disease/disorders from the IPA analysis, stratified by race/ethnicity. (XLS 241 kb)

Additional file 2: **Figure S1.** Venn diagram of overlap in genes and non-coding RNA for three racial/ethnic groups. Figure S2 Regulatory features of cg13986536 in 9q34.11. Figure S3 Regulatory features of cg21842914 in 17q25.3. Figure S4 Regulatory features of cg11413570 in 1p13.3. Figure S5 Regulatory features of cg00812246 in 1p32.3. Figure S6 Regulatory features of cg09168939 in 1q23.3. Figure S7 Regulatory features of cg11108534 in 2p25.2. (PPT 898 kb)

## Abbreviations

*AHRR*: Aryl-hydrocarbon receptor repressor
*ALPPL2*: Alkaline phosphatase genes, placental-like
BMI: Body mass index
ENCODE: Encyclopedia of DNA Elements
EWAS: Epigenome-wide association study
*F2RL3*: Coagulation factor II (thrombin) receptor-like 3 gene
*GPR15*: G protein-coupled receptor
H3K27ac: Histone 3 lysine 27 acetylation
H3K4me1: Histone 3 lysine 4 monomethylation
H3K4me3: Histone 3 lysine 4 trimethylation
H3K9ac: Histone 3 lysine 9 acetylation
NE: Nicotine equivalents
