# Supplementary material for: Association of internal smoking dose with blood DNA methylation in three racial/ethnic populations
Source: Clin Epigenetics. 2018 Aug 23;10:110. doi: 10.1186/s13148-018-0543-7 (PMC6108111; doi:10.1186/s13148-018-0543-7)
Supplement: Supplementary file 2 — Figure S1. Venn diagram of overlap in genes and non-coding RNA for three racial/ethnic groups. Figure S2 Regulatory features of cg13986536 in 9q34.11. Figure S3 Regulatory features of cg21842914 in 17q25.3. Figure S4 Regulatory features of cg11413570 in 1p13.3. Figure S5 Regulatory features of cg00812246 in 1p32.3. Figure S6 Regulatory features of cg09168939 in 1q23.3. Figure S7 Regulatory features of cg11108534 in 2p25.2. (PPT 898 kb) [file 13148_2018_543_MOESM2_ESM.ppt]

## Slide 1
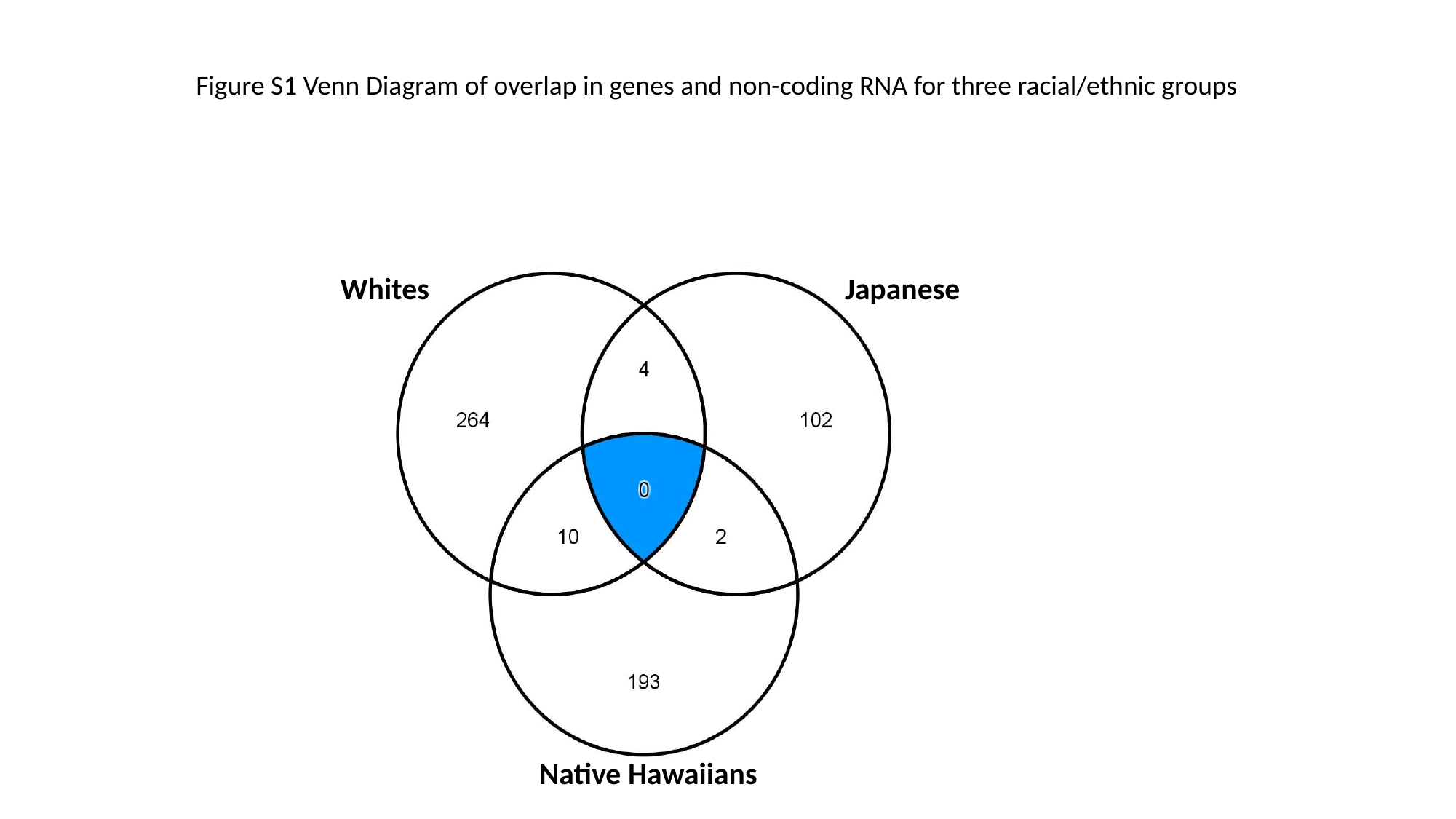

Figure S1 Venn Diagram of overlap in genes and non-coding RNA for three racial/ethnic groups
Whites
Japanese
Native Hawaiians

## Slide 2
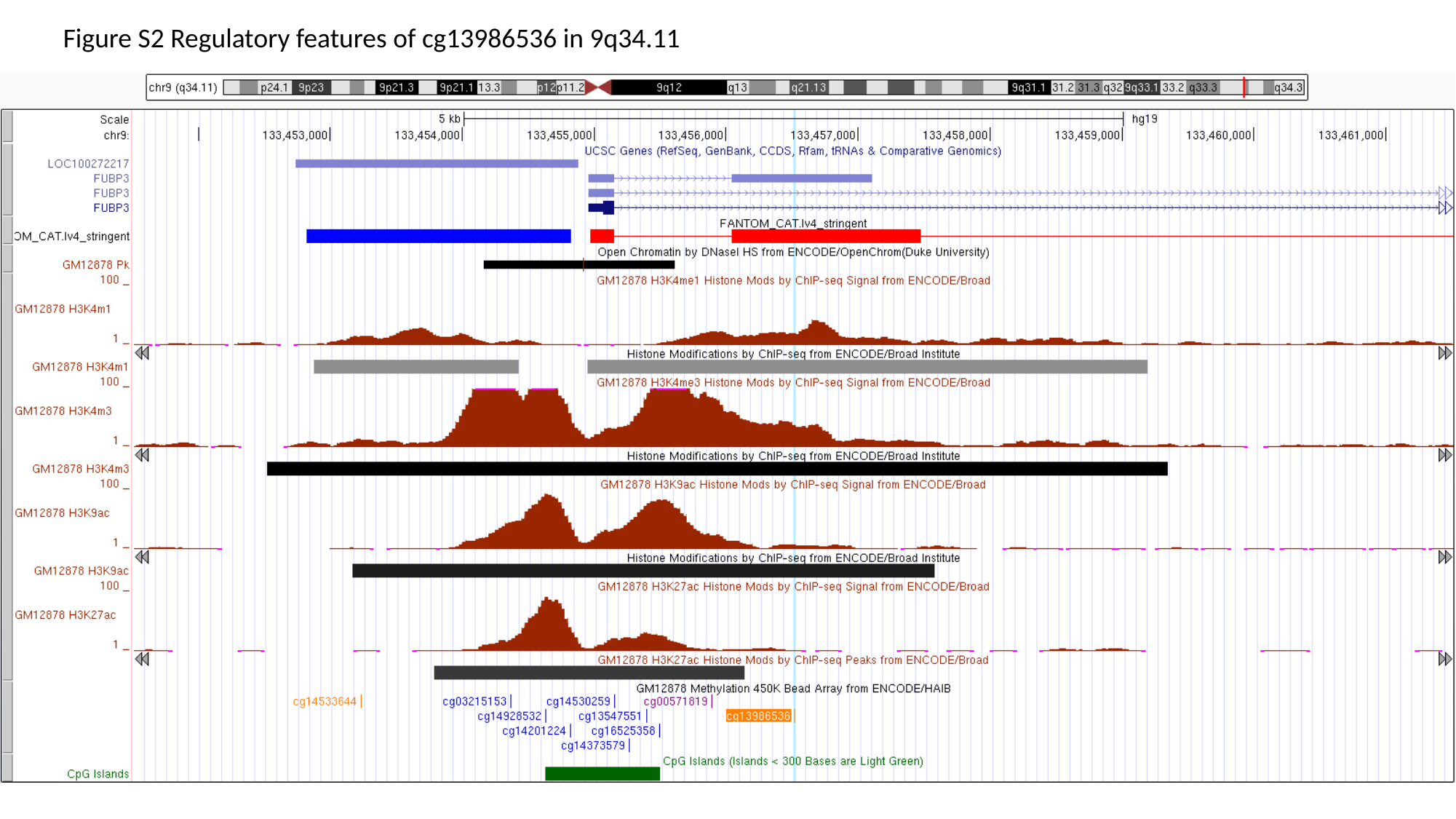

Figure S2 Regulatory features of cg13986536 in 9q34.11

## Slide 3
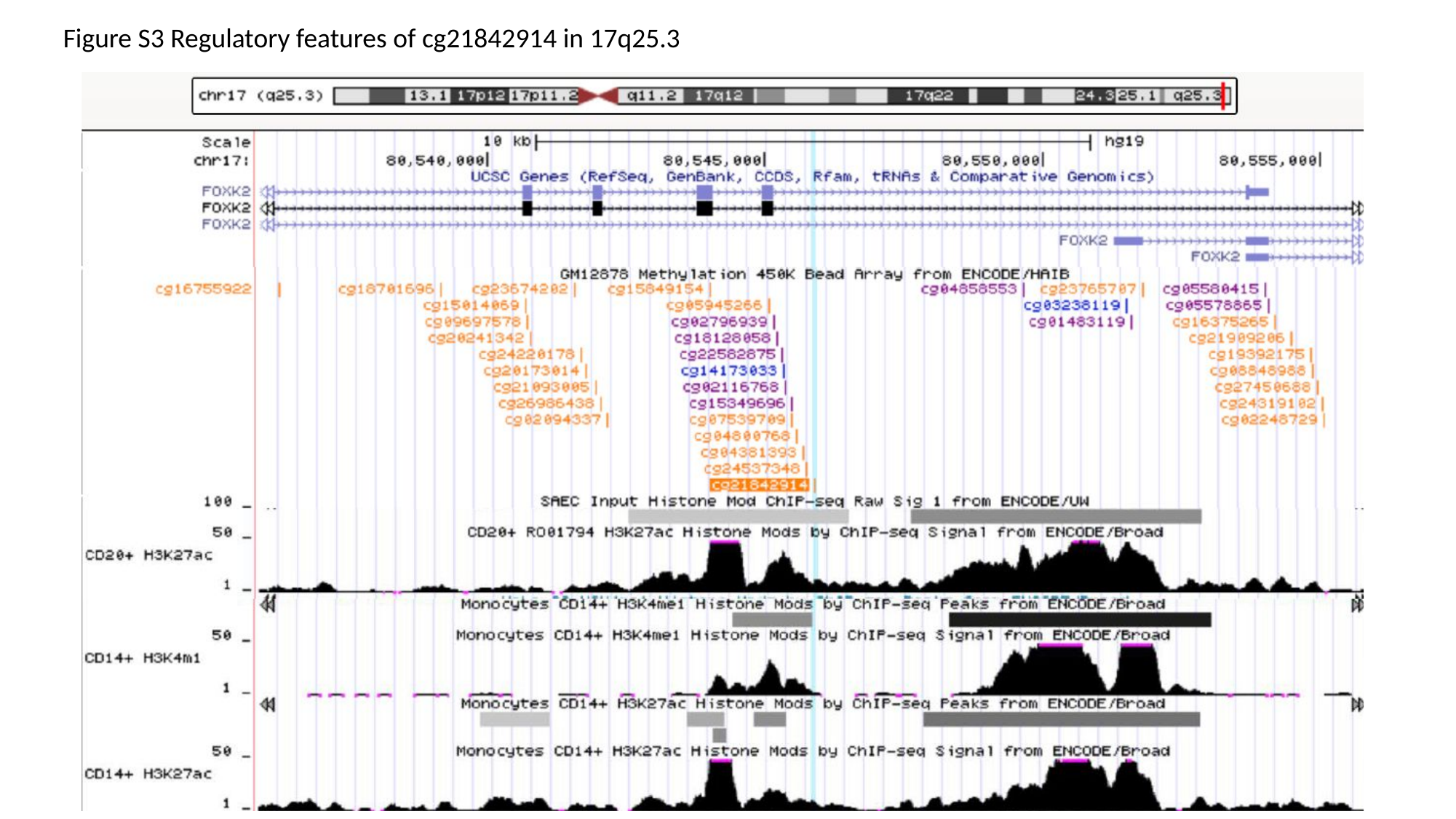

Figure S3 Regulatory features of cg21842914 in 17q25.3

## Slide 4
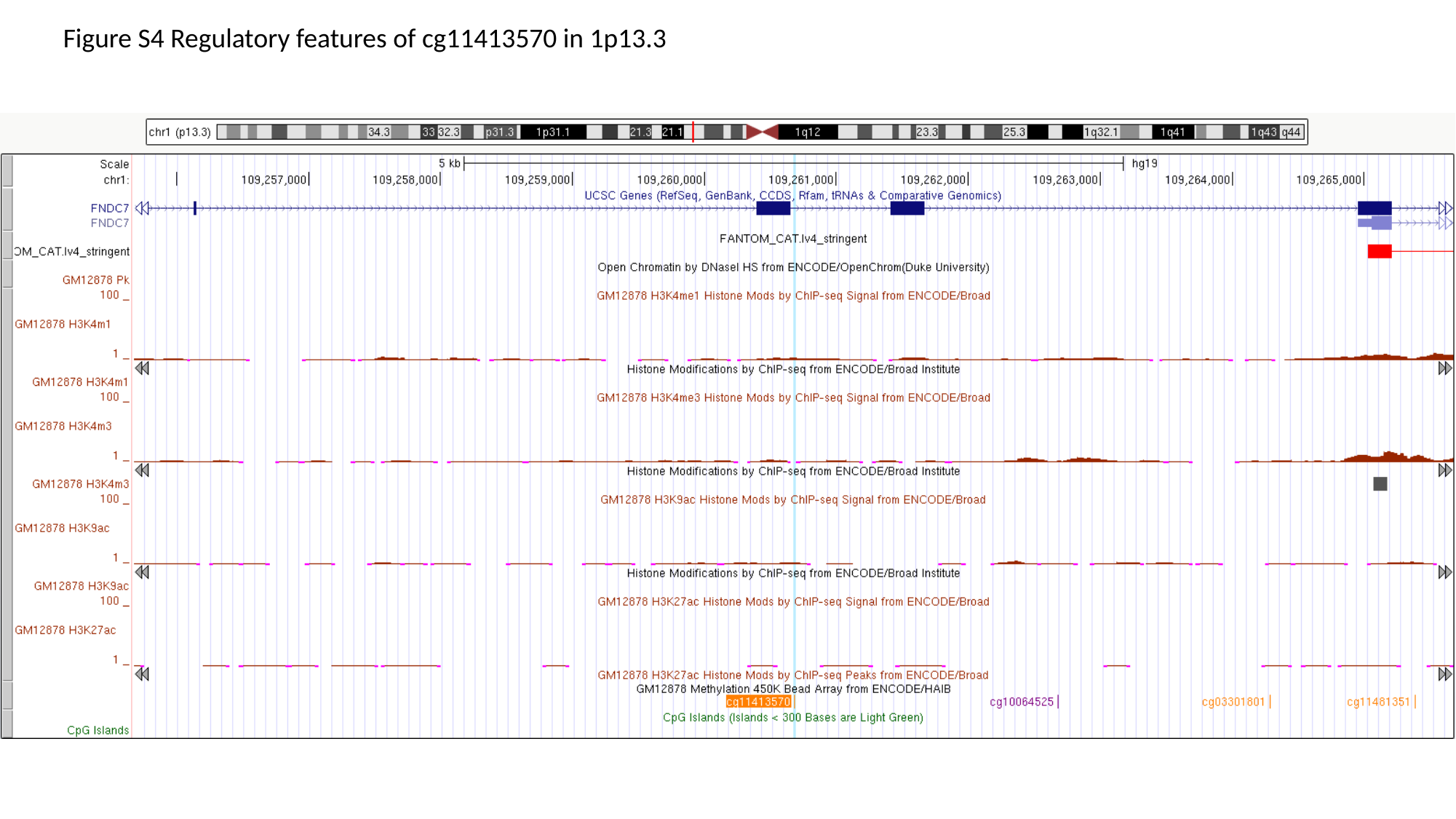

Figure S4 Regulatory features of cg11413570 in 1p13.3

## Slide 5
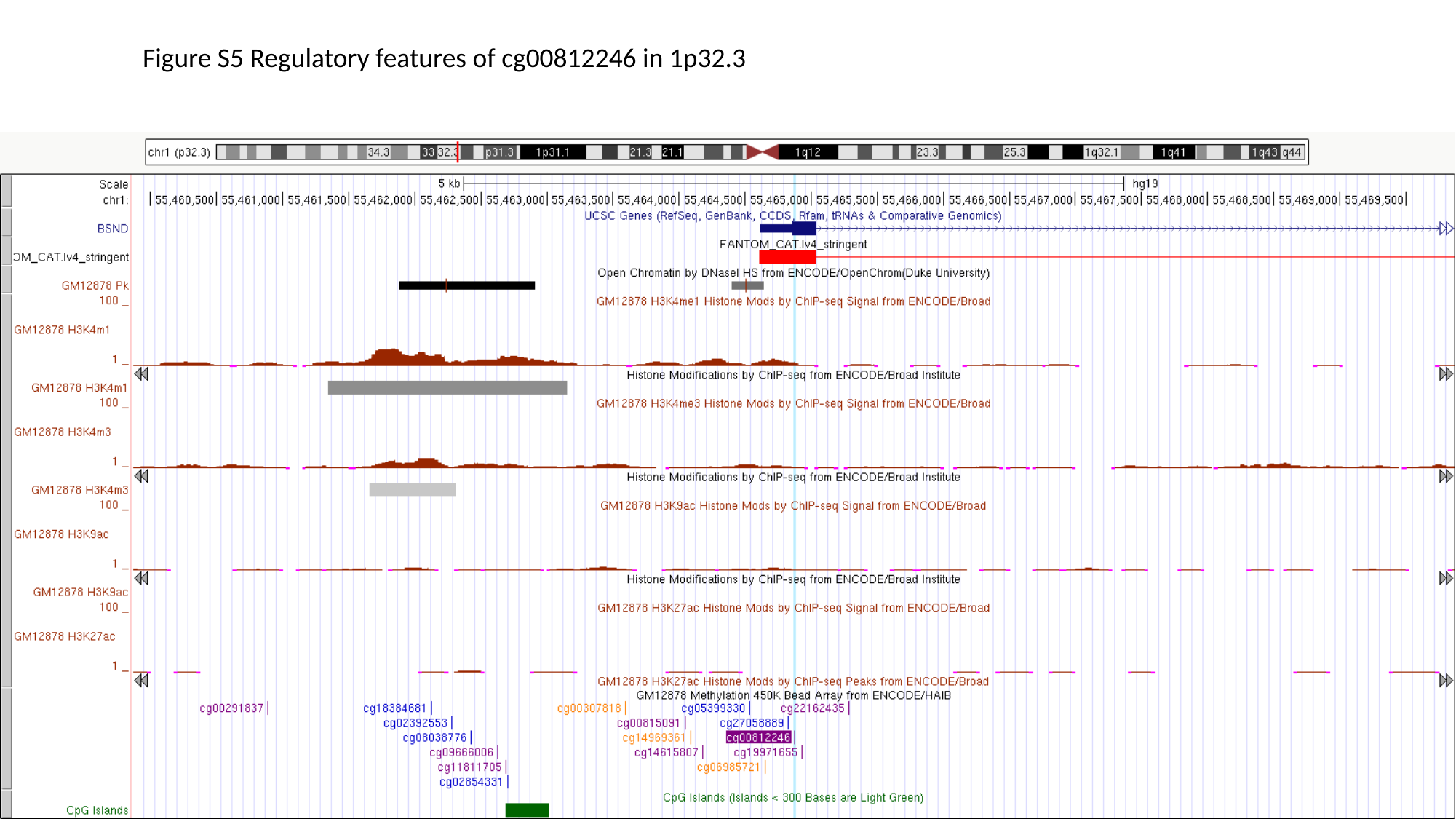

Figure S5 Regulatory features of cg00812246 in 1p32.3

## Slide 6
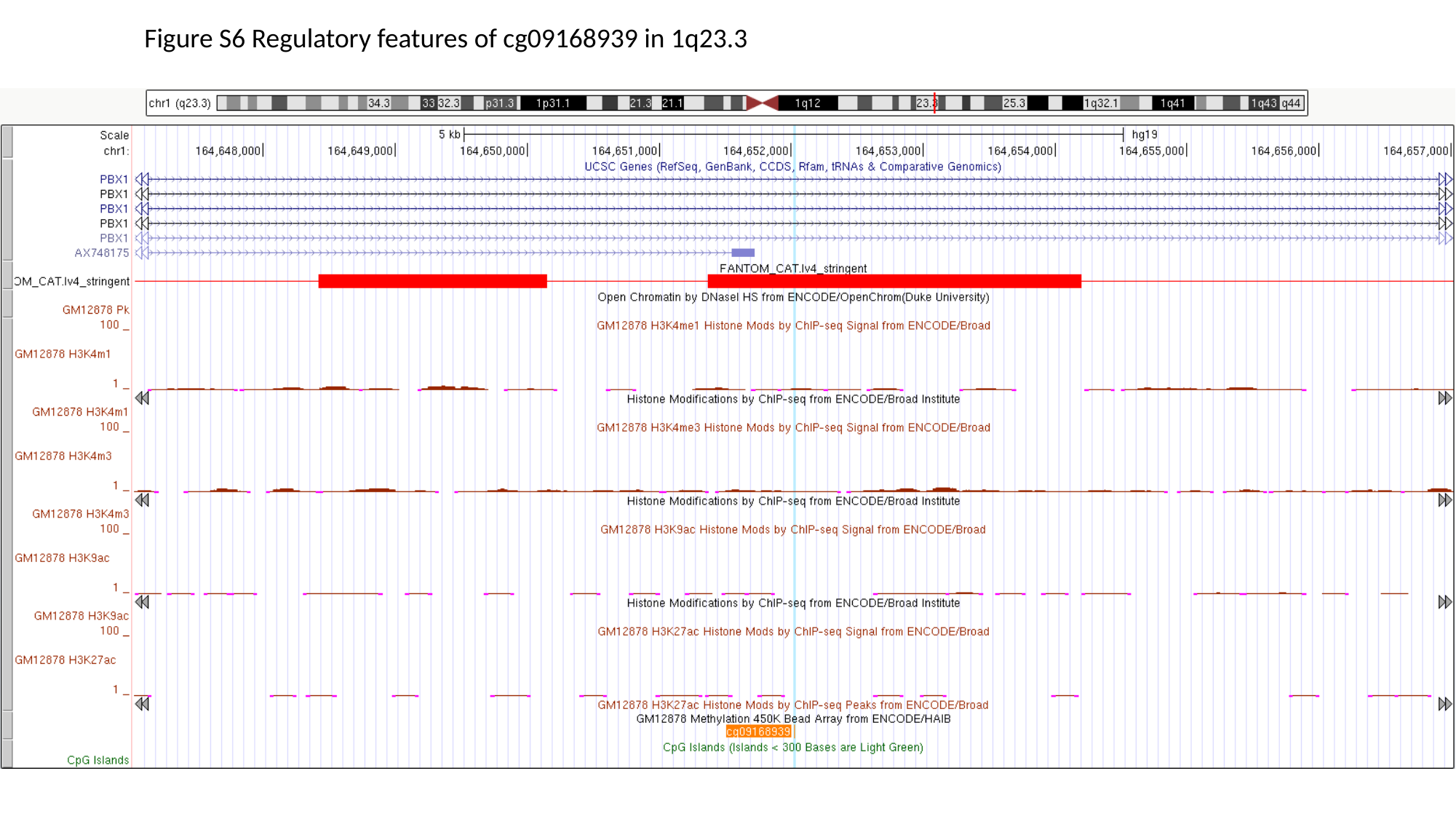

Figure S6 Regulatory features of cg09168939 in 1q23.3

## Slide 7
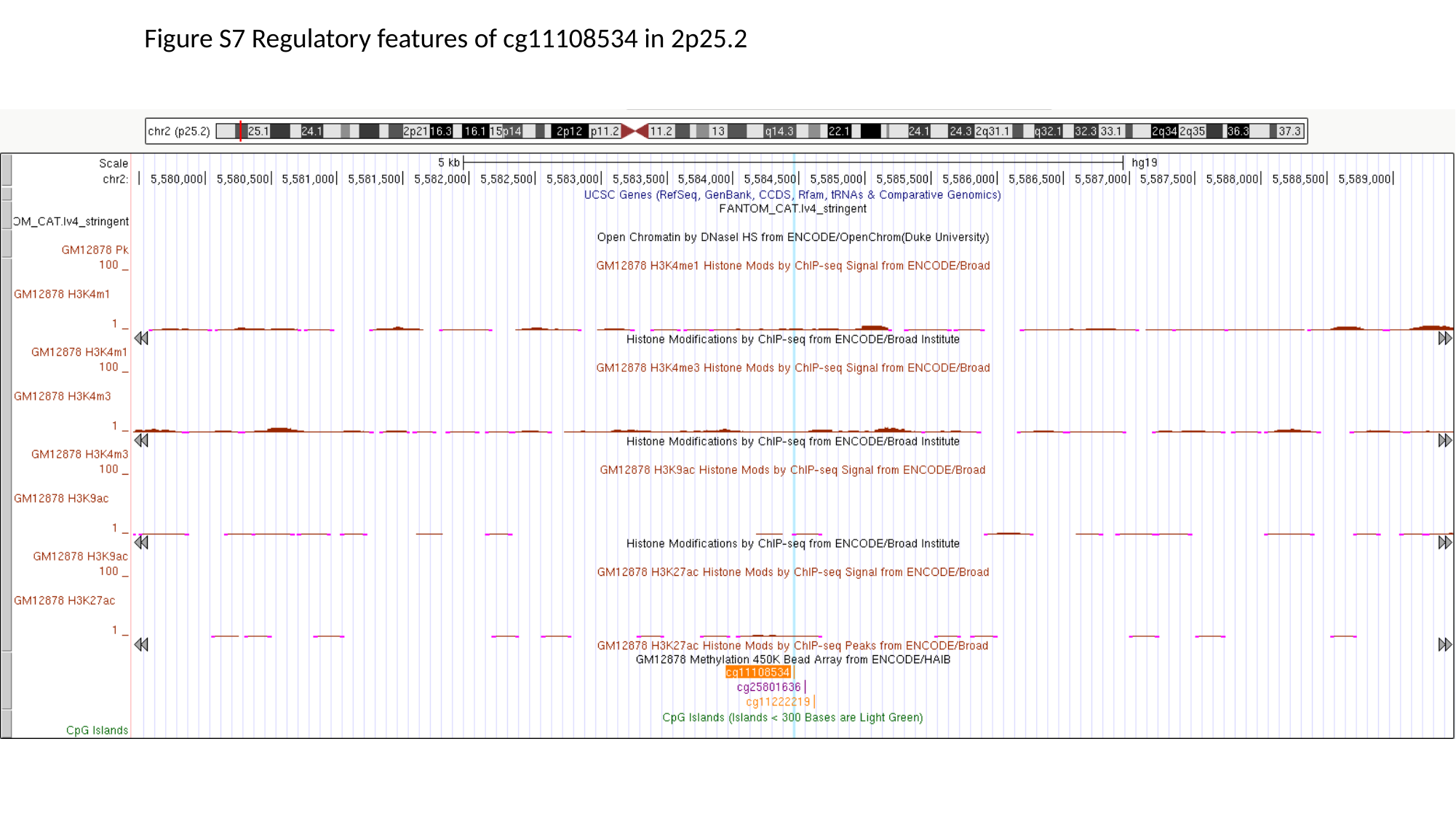

Figure S7 Regulatory features of cg11108534 in 2p25.2
